# Adherence to the Mediterranean Diet and Body Composition in Children and Adolescents with Obesity: A Cross-Sectional Study

**DOI:** 10.3390/nu18213576

**Published:** 2026-10-29

**Authors:** Mirna Begović, Josipa Radić, Marko Šimunović, Marija Selak, Adela Arapović

**Affiliations:** 1 Sestre Milosrdnice University Hospital Center, 10000 Zagreb, Croatia; 2 School of Medicine, University of Split, 21000 Split, Croatiaadela.arapovic@kbsplit.hr (A.A.); 3 Division of Nephrology and Haemodialysis, Department of Internal Medicine, University Hospital of Split, 21000 Split, Croatia; 4 Division of Pulmonology, Allergology, Endocrinology, Cardiology and Rheumatology, Department of Paediatrics, University Hospital of Split, 21000 Split, Croatia; 5 Section of Dietetics, Department of Nutrition and Dietetics, University Hospital of Split, 21000 Split, Croatia; marija.selak@kbsplit.hr; 6 Division of Nefrology, Gastroenterology, Hereditary Metabolism Diseases and Eating Disorders, Department of Paediatrics, University Hospital of Split, 21000 Split, Croatia

**Keywords:** Mediterranean diet, children/adolescent obesity, children/adolescent nutrition, body composition, bioelectrical impedance analysis, KIDMED, anthropometric measurement

## Abstract

**Background/Objectives**: Obesity in children and adolescents remains a major health challenge, while Mediterranean diet (MeDi) adherence among children and adolescents is often suboptimal. This study examined MeDi adherence and its association with anthropometric and body composition parameters in children and adolescents with obesity. **Methods**: This cross-sectional study included 132 participants aged 5–19 years with obesity and was conducted between February 2024 and September 2025 at the Department of Pediatrics, University Hospital Centre Split. MeDi adherence was assessed using the Mediterranean Diet Quality Index for children and adolescents (KIDMED), and body composition by multifrequency bioelectrical impedance analysis. Also, anthropometric measurements were recorded, and body mass index z-score adjusted for age and sex (BMI z-score) was calculated. **Results**: Based on the KIDMED score, 34.8% of participants showed optimal MeDi adherence, 49.2% moderate adherence, and 15.9% very low adherence. Significant inverse correlations were observed between KIDMED scores and body fat mass (kg) (ρ = −0.230, *p* = 0.008) and trunk fat mass (kg) (ρ = −0.234, *p* = 0.007), although these associations did not remain statistically significant after Benjamini–Hochberg correction (adjusted *p* = 0.06 for both). Multivariate linear regression analyses showed no statistically significant associations between KIDMED scores and the examined body fat parameters after adjustment for age and sex (all *p* > 0.05). Age was positively associated with body fat mass (kg), trunk fat mass (kg), and trunk fat (%) (all *p* < 0.001), whereas female sex was associated with higher body fat (%) (*p* < 0.001). **Conclusions**: Adherence to the MeDi was suboptimal in most children and adolescents with obesity. Our findings did not establish a statistically significant association between MeDi adherence and the examined body fat parameters after correction for multiple testing and adjustment for age and sex. Further prospective and intervention studies with a larger number of participants are needed to determine whether improving MeDi adherence can lead to favorable changes in body composition in this population.

## 1. Introduction

The epidemic of overweight and obesity in childhood and adolescence has become one of the most important global public health challenges of the 21st century. According to estimates from the World Health Organization (WHO), more than 390 million children and adolescents aged 5–19 years were overweight in 2022, of whom approximately 160 million were classified as having obesity [1]. Consistent with these global trends, the prevalence of childhood obesity has increased across Europe, including in southern Mediterranean countries [2]. Recent data from the WHO European Childhood Obesity Surveillance Initiative (COSI) confirm a high prevalence of overweight and obesity among children across Europe, including Croatia, with approximately one in four children in Europe (25%) and one in three children in Croatia (36%) affected [3]. An important question remains regarding whether the ongoing epidemic of childhood obesity will lead to an increased risk of cardiovascular (CV) diseases in early adulthood.

Although CV diseases predominantly manifest later in life, atherosclerosis and the CV continuum begin much earlier. Early identification of CV risk factors and subclinical CV alterations during childhood is therefore of particular importance, as these processes may still be amenable to modification at this stage.

Childhood obesity is a multifactorial condition resulting from the interaction of dietary habits, physical inactivity, genetic predisposition, and environmental factors, and is linked to tracking into adulthood [4,5,6]. From a pathophysiological perspective, obesity is a complex metabolic condition characterized by dysregulation of energy homeostasis and glucose, lipid, and adipose tissue metabolism, mediated by central and peripheral mechanisms [4]. Longitudinal studies have shown that higher body mass index (BMI) percentiles in childhood and adolescence predict overweight and obesity in adulthood, highlighting the importance of early lifestyle interventions, as children with obesity are approximately five times more likely to remain obese later in life [7,8].

A healthy and balanced diet, together with lifestyle modifications, represents a key modifiable factor in the prevention of overweight and obesity and, consequently, may play an important role in reducing the burden of chronic non-communicable diseases (NCDs). Childhood adiposity predicts metabolic dysregulation, including insulin resistance and dyslipidemia, in young adulthood [9].

Among various dietary patterns, the Mediterranean diet (MeDi) stands out as a widely recognized model of a healthy diet pattern and has been linked with reduced risk of NCDs, partly through improvements in blood pressure and lipid profile [10,11]. The MeDi is a predominantly plant-based dietary pattern characterized by frequent consumption of fruits, vegetables, legumes, whole grains, and nuts, olive oil as the main source of fat, regular consumption of fish and dairy products, and limited intake of red and processed meat and sweets, thereby providing a rich source of unsaturated fatty acids, fiber, vitamins, and antioxidants [12,13]. Recent evidence indicates that greater adherence to the MeDi is associated with a lower incidence of overweight and obesity in children and with more favourable anthropometric indicators, including lower BMI, waist circumference (WC), and abdominal obesity, already from preschool age [14,15]. In addition to BMI and conventional anthropometric measures, body composition analysis provides information on fat mass and fat-free mass, allowing a more comprehensive assessment of adiposity in children [16]. Findings from intervention studies in overweight children suggest that adherence to the MeDi associated with favourable changes in body composition, including reductions in fat mass with relative preservation of lean mass—changes that may not be adequately captured by BMI alone [17]. A recent meta-analysis of randomized trials found that MeDi–based interventions produce small but significant reductions in BMI and obesity indicators in youth, particularly among those with overweight or obesity [18]. However, an umbrella review highlighted that existing evidence on the association between MeDi adherence and health outcomes in adolescents is limited and heterogeneous, indicating the need for further well-designed studies in this population [19].

Therefore, the aim of this study was to evaluate adherence to the MeDi and its association with anthropometric and body composition parameters in children and adolescents with obesity from the Dalmatian region of Croatia.

## 2. Materials and Methods

### 2.1. Study Design and Population

**Figure 1:**
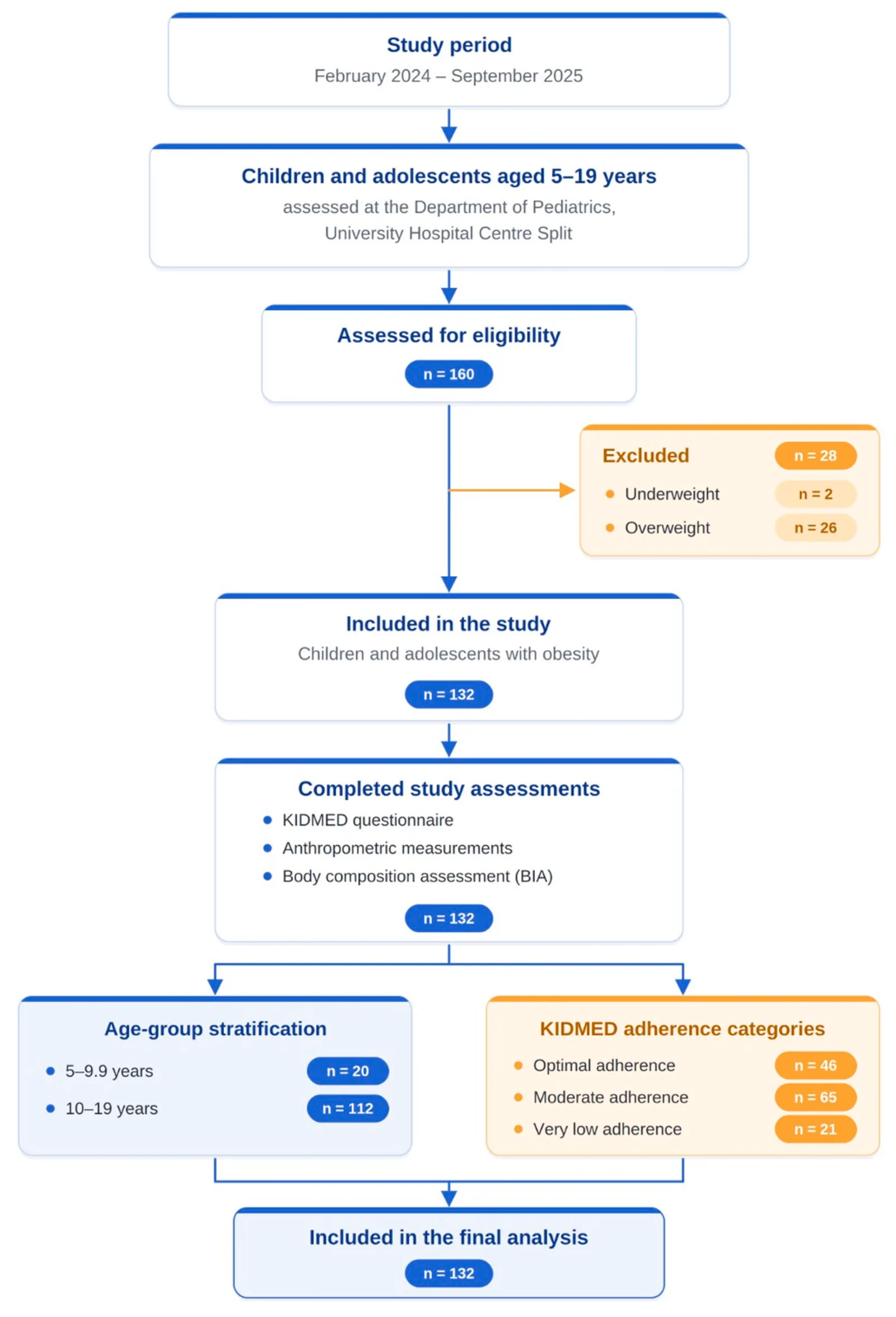
The study flow-chart. Abbreviations: *n*—number.

A total of 132 children with a mean age of 13.31 years, and obesity according to WHO growth charts were included in this cross-sectional study. The study was conducted between February 2024 and September 2025 at the Department of Pediatrics, University Hospital Centre Split. The study flowchart illustrating participant selection from the initial 160 children and adolescents to the final 132 included in the analysis is presented in Figure 1. Parents or legal guardians gave written informed consent and received comprehensive written and spoken information about the study before enrolment. The minimum required sample size was initially estimated at 128 participants, assuming a medium effect size (Cohen’s d = 0.5), a two-sided significance level of 0.05, and a statistical power of 80% for the comparison of two independent groups (G*Power, version 3.1.9.7). As the primary objective was to assess the association between KIDMED scores and body composition parameters, an additional sensitivity power analysis was performed for the final sample of 132 participants. Assuming a two-sided significance level of 0.05 and 80% power, this sample size was sufficient to detect a correlation coefficient of approximately 0.24. Complete KIDMED questionnaire data were available for all 132 participants included in the study. Missing data were not imputed, and analyses were performed using available data for each variable.

For the exploratory age-group comparisons, participants were divided into two groups (5–9.9 and 10–19 years), using 10 years as the cut-off in accordance with the WHO definition of adolescence [20].

Based on this calculation, the following inclusion criteria were applied: children aged 5–19 years classified as obese, without other NCDs, and with written informed consent provided by a parent or legal guardian. The KIDMED questionnaire was completed by parents or legal guardians for younger children, while older children and adolescents who were able to complete it independently did so themselves in the presence of a parent or legal guardian. The same procedure was followed in both cases. Children who refused to participate, had chromosomal abnormalities, had other serious medical conditions that could affect the study outcome, or whose parents did not accurately or completely complete the KIDMED questionnaire were all excluded from the study. The study was approved by the Ethics Committee of the University Hospital of Split on 22 February 2024 (Class: 520-03/24-01/36, Registration No. 2181-147/01-06/LJ.Z.-24-02) and conducted in accordance with the Declaration of Helsinki.

### 2.2. The Mediterranean Diet Quality Index (KIDMED)

Adherence to the MeDi was assessed using the KIDMED. The KIDMED index is based on the principles of the Mediterranean dietary pattern, which is considered one of the healthiest nutritional models, and represents one of the most widely used tools for assessing adherence to this dietary pattern [21]. The original KIDMED questionnaire was administered in Croatian and consisted of 16 “yes” or “no” questions [22]. Its validity has been established in previous studies, and its application in the Croatian population has also been documented [13,23]. For twelve questions reflecting dietary behaviors aligned with the Mediterranean diet, a “yes” response was scored +1 and a “no” response 0. For the four questions reflecting dietary behaviors not aligned with the MeDi (fast-food consumption, skipping breakfast, pastries for breakfast, and consumption of sweets several times per day), a “yes” response was scored −1 and a “no” response 0. The total score ranged from −4 to 12, with higher scores indicating greater adherence to the MeDi. Based on the total score, participants were classified into three categories: (1) ≥8, optimal adherence to the MeDi; (2) 4–7, moderate adherence requiring dietary improvements; (3) ≤3, very low adherence and diet quality [22].

### 2.3. Anthropometric Measurement and Body Composition

Anthropometric parameters were measured in all participants. All measurements were performed by the same examiner under standardized conditions, with participants wearing light clothing and no shoes. All anthropometric values were rounded to one decimal place. Body height (cm) was measured in a standing position, on a firm surface, with the participant’s back aligned and in full contact with the stadiometer, arms relaxed at the sides, heels together, and head positioned straight. Body mass (kg) and BMI (kg/m^2^) were measured with a Tanita MC-780 analyser (Tanita Corporation, Tokyo, Japan).

For each participant, BMI z-scores were calculated using the WHO AnthroPlus software (version 1.0.4), based on the WHO growth reference standards for children and adolescents aged 5–19 years [24]. BMI z-scores express the deviation of an individual’s BMI from the median of the age- and sex-specific reference population, quantified in standard deviation units. This standardized approach accounts for age- and sex-related variations in BMI during growth and development, allowing valid comparisons across pediatric age groups. WHO BMI z-scores are derived from reconstructed age- and sex-specific reference curves, with standard deviation cut-off points aligned with clinically relevant adult BMI thresholds [25]. Accordingly, overweight was defined as a BMI z-score > +1, and obesity as a BMI z-score > +2 [24].

To estimate central fat distribution, waist circumference (WC, cm) and hip circumference (HC, cm) were measured using a non-stretchable, flexible measuring tape, and waist-to-hip ratio (WHR) and waist-to-height ratio (WHtR) were subsequently calculated. WC was measured in a standing position, horizontally around the trunk at the level of the upper lateral border of the iliac crest along the mid-axillary line, at the end of normal expiration, whereas HC was measured at the widest circumference of the buttocks [26].

Body composition was assessed using the Tanita MC-780 analyser, a multi-frequency segmental body composition analyser employing bioelectrical impedance analysis (BIA) [27,28]. Although BIA has been used for body composition assessment in pediatric populations with obesity or high body weight, its accuracy may be affected by the degree of adiposity, and limitations have been reported for segmental body composition assessment [27]. BIA measurements were performed during the morning hours, with participants barefoot and wearing light clothing. Parents were advised that children should preferably attend the assessment in a fasting state or at least two hours after food and fluid intake, when possible. However, compliance with these recommendations was not systematically verified. Body composition parameters obtained from the analyser included fat mass (kg), body fat percentage (%), fat-free mass (FFM, kg), muscle mass (kg), phase angle (PhA, °), impedance (Ohm), trunk fat mass (kg), trunk fat percentage (%), trunk fat-free mass (kg), trunk muscle mass (kg), total body water (TBW, kg), and skeletal muscle index (SMI). SMI was calculated as skeletal muscle mass (kg) divided by height squared (m^2^) and was used as an indicator of relative skeletal muscle mass. PhA was derived from resistance and reactance values obtained by BIA and was used as an indicator of the electrical properties of body tissues, reflecting cellular integrity and body cell mass.

### 2.4. Statistical Analyses

Categorical variables are presented as absolute and relative frequencies, while continuous variables are presented as medians and interquartile ranges (IQR). Normality of continuous variables was assessed with the Shapiro–Wilk test. Differences in categorical variables between groups were assessed using the chi-square test. Differences in continuous variables between two independent groups were assessed using the Mann–Whitney U test. Comparisons of continuous variables among the three KIDMED categories were performed using the Kruskal–Wallis test, followed by Conover’s post hoc test for pairwise comparisons when an overall statistically significant difference was observed.

The association between continuous KIDMED scores, anthropometric indices and body composition parameters was assessed by Spearman’s rank correlation coefficient (ρ). Partial correlations adjusted for age and sex were calculated using a rank-based approach.

Multivariate linear regression analyzes were performed to assess the association between continuous KIDMED scores and body composition parameters. Separate models were created for fat mass (kg), trunk fat mass (kg), body fat percentage, and trunk fat percentage. Each model included KIDMED score, age, and sex as independent variables, entered simultaneously using the Enter method. Model fit was assessed using the coefficient of determination (R^2^), adjusted R^2^, and the F statistic. Regression diagnostics included assessment of residuals, multicollinearity and influential observations. Sensitivity analyses were conducted by excluding observations with a Cook’s distance greater than 4/n.

The Benjamini–Hochberg procedure was applied separately to correlation, Kruskal–Wallis, and regression analyses to control the false discovery rate at 5%. Unadjusted and adjusted *p* values are reported where applicable. All *p* values were two-sided, and the significance level was set at α = 0.05. Statistical analyses were performed using IBM SPSS Statistics, version 25.0 (IBM Corp., Armonk, NY, USA) and MedCalc Statistical Software, version 23.6.5 (MedCalc Software Ltd., Ostend, Belgium).

## 3. Results

A total of 132 children and adolescents with obesity, classified according to the WHO BMI-for-age z-score criteria, were included in the study. The study population comprised 68 (51.5%) boys and 64 (48.5%) girls, with most participants aged 10–19 years (84.8%). The median age was 13.31 years (IQR 11.25–15.91), with no statistically significant difference between boys and girls (Mann–Whitney U test, *p* = 0.71). The anthropometric and body composition parameters, together with KIDMED scores, are presented in Table 1.

Assessment of dietary habits using the KIDMED questionnaire showed that 71.2% of participants consumed fruit or fruit juice daily, while 72.7% reported daily consumption of vegetables. However, only 42.4% consumed a second serving of fruit during the day, and 24.2% consumed vegetables more than once daily. Regular fish consumption (at least 2–3 times per week) was reported in 25% of participants, whereas 46.2% consumed legumes more than once weekly. Most participants consumed cereals or cereal products for breakfast (81.8%) and dairy products at breakfast (75.8%). Olive oil was used in meal preparation in 88.6% of households. On the other hand, 41.7% of participants skipped breakfast, 47.7% consumed pastries or baked goods for breakfast, and 41.7% reported consuming sweets several times daily.

According to the KIDMED score, 34.8% of participants had optimal adherence to the MeDi, 49.2% required improvements in dietary habits to better adhere to the Mediterranean dietary pattern, while 15.9% had very low diet quality.

**Figure 2:**
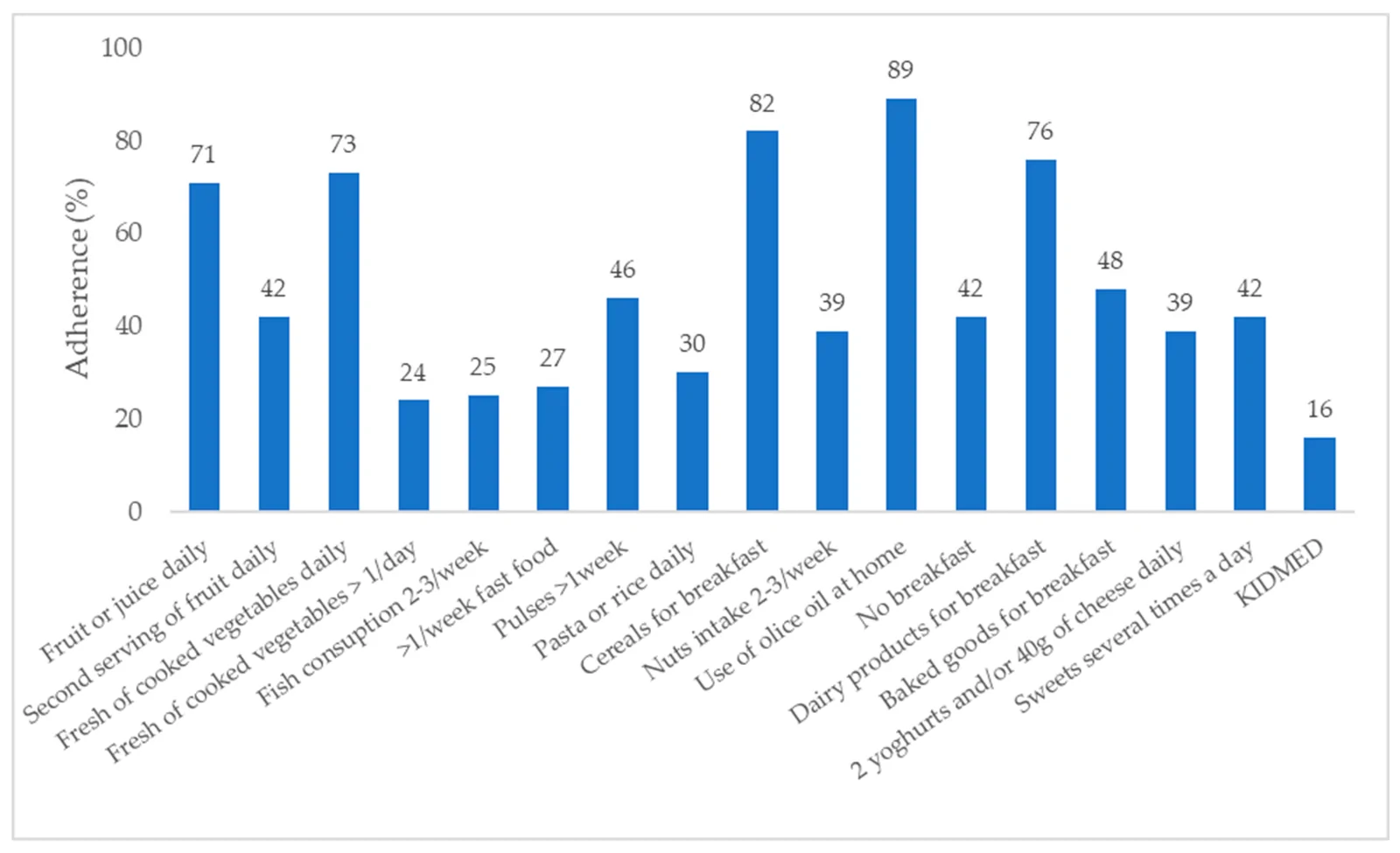
Adherence to the Mediterranean diet and its components for the total study population.

The distribution of responses to individual items of the KIDMED questionnaire is presented in Figure 2. The highest proportions of “yes” responses were related to the use of olive oil at home, cereal consumption at breakfast, and dairy product consumption at breakfast, whereas regular fish consumption and frequent vegetable intake were less commonly reported.

Differences in anthropometric and body composition parameters across the three KIDMED categories are presented in Supplementary Table S1. Age was the only variable showing a nominally significant difference between groups (Kruskal–Wallis test, *p* = 0.03), with participants in the very low diet quality group being older than those requiring dietary improvements (median age: 15 vs. 13 years). However, this difference was no longer statistically significant after Benjamini–Hochberg correction. No significant differences in anthropometric or body composition parameters were observed across KIDMED categories.

**Figure 3:**
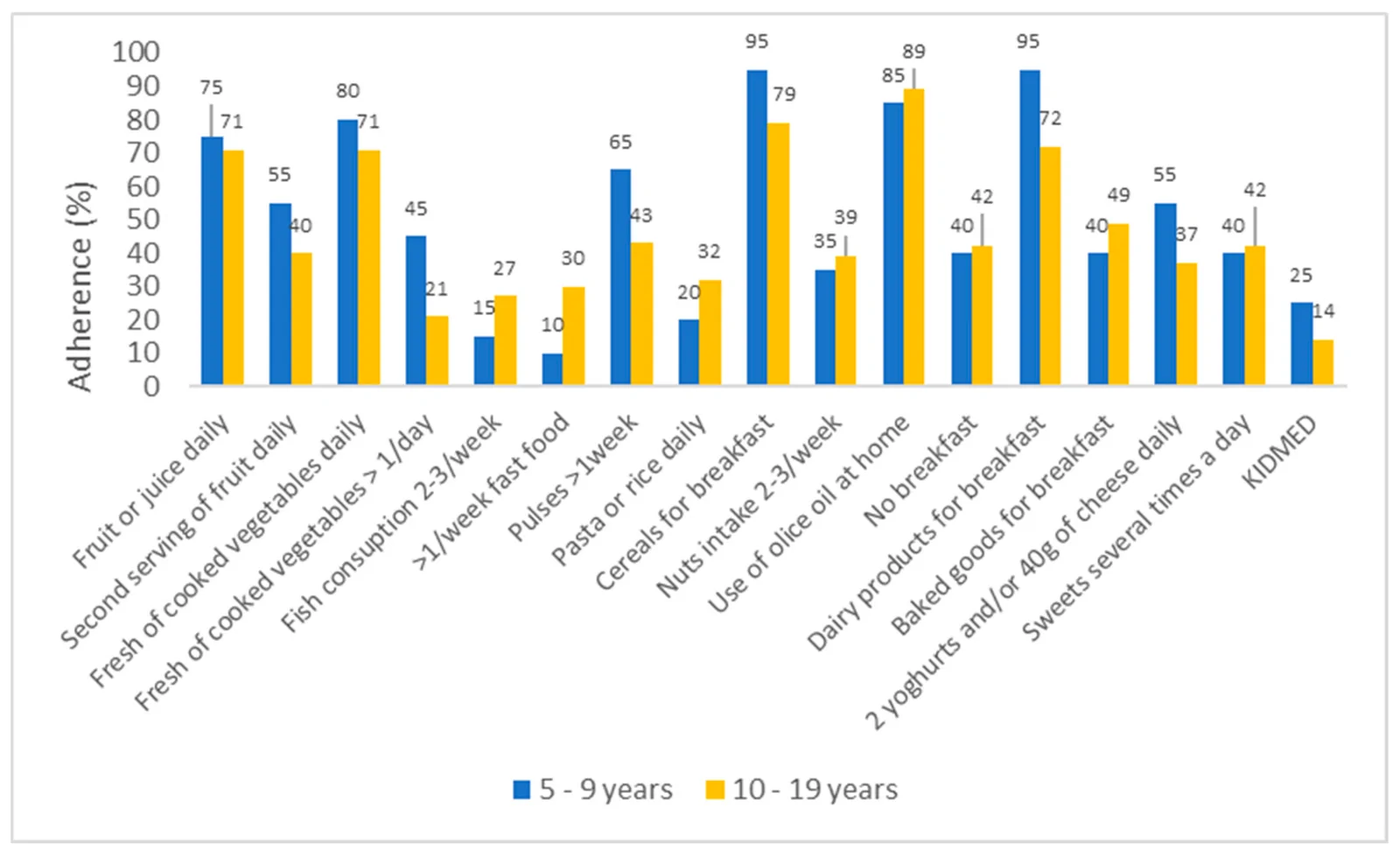
Adherence to the Mediterranean diet and its components regarding two age groups.

Age-group comparisons of individual KIDMED dietary recommendations showed that children aged 5–9.9 years more frequently consumed vegetables more than once daily than those aged 10–19 years (45% vs. 21%, Chi-square test, *p* = 0.02). Similarly, dairy product consumption at breakfast was more frequent among younger children (95% vs. 72%, Chi-square test, *p* = 0.04). These age-group comparisons were exploratory and were not adjusted for multiple testing; therefore, the nominally significant findings should be interpreted with caution (Figure 3 and Supplementary Table S2).

Table 2 presents unadjusted Spearman’s rank correlations and partial rank correlations adjusted for age and sex between KIDMED scores and anthropometric and body composition parameters. Weak negative correlations were observed between KIDMED scores and fat mass (ρ = −0.230, *p* = 0.008) and trunk fat mass (ρ = −0.234, *p* = 0.007); however, neither association remained statistically significant after Benjamini–Hochberg correction (adjusted *p* = 0.06 for both). A weak positive partial correlation was observed between KIDMED scores and PhA (ρ = 0.196, *p* = 0.03), but this association also did not remain significant after correction (adjusted *p* = 0.51). No statistically significant associations were observed after adjustment for age and sex and correction for multiple testing.

Sensitivity analyses were performed after excluding observations with extreme BMI z-score (5.81) and PhA (10.9°) values. Exclusion of the extreme BMI z-score yielded virtually unchanged unadjusted (ρ = 0.007, *p* = 0.94) and age- and sex-adjusted partial correlations (ρ = −0.044, *p* = 0.62). Similarly, exclusion of the extreme PhA value resulted in an unadjusted correlation of ρ = 0.107 (*p* = 0.23) and an adjusted partial correlation of ρ = 0.216 (*p* = 0.01). These sensitivity analyses were conducted to assess the robustness of the primary findings without additional multiple-testing correction. The magnitude and direction of the associations remained largely unchanged, and the overall conclusions were unaffected.

Multivariate linear regression analyses were performed to assess the association between KIDMED scores and body composition parameters after adjustment for age and sex. KIDMED scores were not significantly associated with fat mass, trunk fat mass, body fat percentage, or trunk fat percentage in any of the adjusted models.

Age was positively associated with fat mass, trunk fat mass, and trunk fat percentage (all *p* < 0.001), while female sex was positively associated with body fat percentage (*p* < 0.001). No associations between KIDMED scores and body composition parameters remained significant after Benjamini–Hochberg correction. No evidence of multicollinearity was observed (VIF range: 1.006–1.055) (Table 3).

## 4. Discussion

Although the MeDi represents the traditional dietary pattern of the study region, only 34.8% of participants achieved optimal adherence according to the KIDMED index, whereas the majority required improvements in their dietary habits. These findings are consistent with previous studies conducted among children and adolescents in Mediterranean European countries [29]. A systematic review by Iaccarino Idelson et al. demonstrated that adherence to the MeDi among children and adolescents is frequently suboptimal, even in countries where this dietary pattern traditionally predominates [21]. Likewise, Grosso and Galvano highlighted a progressive shift from traditional Mediterranean eating habits towards more Westernized dietary patterns among younger generations in Southern Europe [30]. Similar trends have also been observed in Croatia. Sila et al. reported suboptimal adherence to the MeDi among Croatian schoolchildren, with significant geographic differences and generally better adherence in Mediterranean than in continental regions [31]. These findings were further supported by more recent Croatian data showing predominantly low-to-moderate adherence among children aged 2–9 years, with higher KIDMED scores observed in younger children and those living in coastal regions, particularly Dalmatia and Istria, as well as in the Zagreb area, suggesting a potential influence of regional and socioeconomic factors on dietary habits [32]. Furthermore, Matana et al. reported generally low adherence among Croatian children and adolescents and emphasized that the traditional Mediterranean dietary pattern is increasingly being abandoned even in Mediterranean regions of Croatia [33]. These observations are consistent with local data from Split-Dalmatia County, where predominantly low adherence was also reported among preschool children [13].

Although previous studies have identified several demographic, socioeconomic, and lifestyle factors associated with adherence to the MeDi, evidence specifically focusing on children and adolescents with obesity remains limited. In the present study, participants with very low diet quality were generally older than those requiring dietary improvements, although this difference was not statistically significant after correction for multiple comparisons. Similar age-related trends have been reported in the general pediatric population. Data from the Portuguese Eat Mediterranean Program showed that adherence to the MeDi was lowest among adolescents and older participants aged 15–21 years, whereas the highest adherence was observed in children aged 6–9 years [34]. This possible age-related decline in adherence may be related to increasing dietary autonomy during adolescence, reduced parental influence on food choices, and greater exposure to energy-dense, nutrient-poor foods, all of which have been associated with poorer diet quality in this age group. In this context, differences in individual dietary habits were also observed between the age groups, with younger children (5–9.9 years) more frequently consuming vegetables more than once daily and dairy products at breakfast than older participants (10–19 years). However, these findings should be interpreted cautiously given the exploratory nature of the analyses and the unequal age-group sizes.

Regarding body composition, KIDMED scores showed weak inverse correlations with body fat mass and trunk fat mass, although these associations did not remain statistically significant after correction for multiple testing. The correlations were attenuated after adjustment for age and sex, suggesting that the initial associations may have been partly influenced by these factors. Consistent with these findings, multivariable linear regression analyses did not demonstrate statistically significant associations between KIDMED scores and the examined body fat parameters. Similarly, no significant differences in anthropometric or body composition parameters were observed across KIDMED adherence categories. The characteristic composition of the MeDi, including a high intake of fiber-rich plant foods and unsaturated fats, may contribute to favourable metabolic outcomes. Such a dietary pattern has been associated with greater satiety, lower dietary energy density, improved insulin sensitivity, and lower adiposity [35,36]. Previous studies examining MeDi adherence and obesity-related outcomes in pediatric populations have reported mixed findings. Manzano-Carrasco et al. found no significant differences in total fat mass or trunk fat mass across KIDMED adherence categories in Spanish children and adolescents stratified by sex and pubertal status [37]. In contrast, Mistretta et al. reported inverse associations between KIDMED scores and BMI, WC, and fat mass among Southern Italian adolescents [38]. Furthermore, two large cross-sectional studies conducted among Greek children aged 2–5 and 6–9 years reported associations between lower MeDi adherence and a higher prevalence of overweight, obesity, and abdominal obesity [14,15]. Evidence from intervention studies provides additional context. A systematic review and meta-analysis of randomized controlled trials found that MeDi-based interventions were associated with small but statistically significant reductions in BMI and obesity prevalence among children and adolescents, whereas the pooled effects on WC, WHtR, and abdominal obesity prevalence were not statistically significant [18]. However, unlike many previous studies conducted in broader pediatric populations, our cohort included only children and adolescents with obesity. This difference in study populations should be considered when interpreting and comparing the findings. In addition to adiposity-related measures, we examined the association between MeDi adherence and PhA, a bioelectrical impedance parameter that provides complementary information on tissue properties and body composition [39]. Although a weak positive correlation with KIDMED scores was observed after adjustment for age and sex, this association did not remain statistically significant after Benjamini–Hochberg correction. Previous studies in adolescents have shown that PhA is associated with age, sex, biological maturation, and body composition [40,41]. These findings highlight the importance of considering developmental and body composition-related factors when interpreting PhA in pediatric populations. Therefore, our findings do not provide sufficient evidence of an association between MeDi adherence and PhA in children and adolescents with obesity. The multivariable linear regression analyses showed different patterns of association between age, sex, and the examined body fat parameters. Age was positively associated with fat mass (kg), trunk fat mass (kg), and trunk fat (%), whereas no significant association was observed with body fat (%). These findings highlight the importance of distinguishing between absolute and relative measures of adiposity during childhood and adolescence. Kaczmarek et al. reported that absolute fat mass increased with age in both sexes among Polish adolescents with normal weight, whereas body fat (%) decreased in boys and increased in girls [42]. Although their study population differed from ours, these findings illustrate that age-related patterns may vary according to the body composition parameter examined. In our study, female sex was significantly associated with higher body fat (%), whereas no significant association was observed with fat mass (kg) in the multivariable regression model. This pattern may partly reflect sex-related differences in FFM, as body fat (%) depends on the proportion of fat mass (kg) relative to total body mass (kg). Kasović et al. reported higher FFM in boys than in girls in a large sample of Croatian adolescents assessed using BIA [43]. Such sex-related differences in FFM may be partly related to pubertal development, during which boys typically experience a greater increase in FFM than girls, contributing to sex-specific patterns of body composition [44]. Notably, age was positively associated with both trunk fat mass (kg) and trunk fat (%), whereas sex was not significantly associated with either parameter. The association between age and trunk fat (%) was observed despite the absence of a statistically significant association with body fat (%). These findings suggest that age should be considered when interpreting trunk fat parameters in children and adolescents with obesity.

The present study has several limitations. First, the study had a cross-sectional design, limiting causal inference. In addition, adherence to the MeDi was assessed using the KIDMED questionnaire, which may be subject to reporting bias. Although the Croatian version of the questionnaire has been used in previous studies, its formal linguistic and psychometric validation could not be confirmed, representing an additional methodological limitation. Furthermore, although the questionnaire was completed by parents or legal guardians for younger children and independently by older children and adolescents, respondent type was not systematically recorded at the individual level. Therefore, potential differences between parent-proxy and self-reported responses should be considered when interpreting the observed age-related differences in KIDMED scores. Information on physical activity, pubertal status, and socioeconomic characteristics was not available in this study. The lack of information on pubertal status is particularly relevant given the broad age range of the participants and the potential influence of growth and pubertal development on dietary behavior and body composition. Although pre-measurement recommendations were provided, strict fasting and hydration conditions were not enforced, and adherence was not systematically verified. These factors may have influenced the BIA-derived body composition estimates. Furthermore, the unequal sizes of the younger (5–9.9 years; *n* = 20) and older (10–19 years; *n* = 112) age groups limit the interpretation of age-group comparisons. The relatively small sample size, particularly within specific subgroups, may also have limited statistical power to detect associations. Furthermore, the original sample size calculation was based on a comparison between two independent groups rather than being specifically designed for the primary regression analyses. Examining multiple, closely related body composition parameters increased the number of statistical comparisons. Although the Benjamini–Hochberg procedure was applied to address multiple testing, the findings should be interpreted cautiously, particularly given the correlations among BIA-derived outcomes. Finally, as most participants were recruited from the Split-Dalmatia region, the generalizability of the findings to other pediatric populations may be limited.

Despite these limitations, the study provides additional information on MeDi adherence and body composition in children and adolescents with obesity. The use of body composition measures derived from BIA, combined with age- and sex-adjusted analyses and correction for multiple comparisons, enabled a more comprehensive assessment of the associations under study. Further prospective studies with larger sample sizes and detailed information on development, physical activity, socioeconomic characteristics, and energy intake are needed to clarify the potential link between adherence to the MeDi and body composition in children and adolescents with obesity.

## 5. Conclusions

As diet represents a key modifiable determinant of childhood obesity, increasing attention has been directed toward dietary patterns with established cardiometabolic benefits in this specific population. Because dietary habits are formed early in life and nutrition plays an important role in growth and development, early childhood represents a critical period for establishing healthy eating patterns with long-term health implications. In the present study, adherence to the MeDi was suboptimal in the majority of children and adolescents with obesity. We examined the associations between KIDMED scores and anthropometric and body composition parameters. Although weak inverse correlations were observed between KIDMED scores and fat mass (kg) and trunk fat mass (kg), these associations did not remain statistically significant after correction for multiple testing. Furthermore, multivariable regression analyses showed no statistically significant associations between KIDMED scores and the examined body fat parameters after adjustment for age and sex, whereas age and sex were significantly associated with some of these parameters.

The MeDi remains a valuable dietary pattern for promoting healthy eating habits in children and adolescents. Future prospective and intervention studies with a larger number of participants are needed to determine whether improving adherence to the MeDi leads to improvements in body composition, and long-term cardiometabolic outcomes.

## Figures and Tables

**Table 1:** Basic characteristics, anthropometric measurements, body composition parameters, and KIDMED scores of the study population (*n* = 132).

	Median (IQR)	Range (Min–Max)
Age (years)	13.31 (11.25–15.91)	5.68–18.69
Age of boys (*n* = 68)	13.3 (11.6–15.0)	5.7–18.1
Age of girls (*n* = 64)	13.4 (10.6–16.4)	6.1–18.7
WHR	0.9 (0.8–0.9)	0.7–1
WHtR	0.57 (0.53–0.61)	0.48–0.75
Body weight (kg)	83 (67–96.63)	30.5–147.9
Body height (cm)	164.4 (154.13–173.65)	120.5–194
BMI z-score	2.8 (2.36–3.28)	2.01–5.81
Body fat (%)	34.45 (31–39.03)	18.1–50.6
Fat mass (kg)	27.55 (22.4–36.78)	9.8–60.2
FFM (kg)	52.75 (42.9–64.25)	20.7–90.7
TBW (kg)	38 (31.43–44.95)	15.2–66.4
Muscle mass (kg)	50.25 (40.73–61.25)	19.6–84.1
SMI	8.25 (7.41–9.28)	5.03–12.99
Impedance (Ohm)	574 (539–622.5)	392–792
PhA (°)	5.6 (5.3–6.1)	4.3–10.9
Trunk fat %	30.45 (26.18–36.08)	17.9–53
Trunk fat mass (kg)	12.1 (9.93–17.33)	4.6–37.9
Trunk fat-free mass (kg)	29.55 (23.83–34.6)	13.2–45.6
Trunk muscle mass (kg)	28.2 (22.53–33.03)	12.3–43.5
KIDMED	5 (3–7)	−1–11

Abbreviations: IQR—interquartile range; WHR—waist-to-hip ratio; WHtR—waist-to-height ratio; BMI—body mass index; FFM—fat-free mass; TBW—total body water; SMI—skeletal muscle index; PhA—phase angle; KIDMED—Mediterranean Diet Quality Index for Children and Adolescents.

**Table 2:** Unadjusted and age- and sex-adjusted correlations between KIDMED score and anthropometric and body composition parameters (*n* = 132).

	Spearman’s ρ	*p* Value	*p* Adjusted (BH)	Partial Rank Correlation (ρ)	*p* Value	*p* Adjusted (BH)
PhA (°)	0.095	0.28	0.36	0.196	0.03	0.51
BMI z-score	0.008	0.93	0.93	−0.047	0.60	0.97
Age (years)	−0.213	0.01	0.06	-	-	-
WHR	0.038	0.67	0.71	−0.037	0.68	0.97
WHtR	−0.098	0.26	0.36	−0.091	0.31	0.88
Body weight (kg)	−0.184	0.03	0.14	−0.051	0.57	0.97
Body height (cm)	−0.147	0.09	0.16	−0.030	0.73	0.97
Body fat (%)	−0.139	0.11	0.17	−0.104	0.24	0.82
Fat mass (kg)	−0.230	0.008	0.06	−0.124	0.16	0.82
FFM (kg)	−0.143	0.10	0.16	0.004	0.96	0.98
TBW (kg)	−0.148	0.09	0.16	−0.039	0.66	0.97
Muscle mass (kg)	−0.164	0.06	0.15	−0.044	0.62	0.97
SMI	−0.087	0.32	0.38	−0.008	0.93	0.98
Impedance (Ohm)	0.056	0.52	0.59	−0.029	0.74	0.97
Trunk fat (%)	−0.182	0.04	0.14	−0.112	0.20	0.82
Trunk fat mass (kg)	−0.234	0.007	0.06	−0.116	0.19	0.82
Trunk fat-free mass (kg)	−0.143	0.10	0.16	−0.005	0.96	0.98
Trunk muscle mass (kg)	−0.161	0.06	0.15	−0.003	0.98	0.98

Abbreviations: PhA—phase angle; WHR—waist-to-hip ratio; WHtR—waist-to-height ratio; BMI—body mass index; FFM—fat free mass; SMI—skeletal muscle index; TBW—total body water; BH—Benjamini–Hochberg. Partial rank correlations were adjusted for age and sex; age was therefore excluded from the partial correlation analysis. *p* values were adjusted for multiple comparisons using the Benjamini–Hochberg procedure, and assessed separately for Spearman’s and partial correlations.

**Table 3:** Multivariate linear regression analyses of the association between KIDMED score.

Outcome/Predictors	B (95% CI)	β	*p* Value	*p* Adjusted (BH)
Fat mass (kg)				
KIDMED score	−0.360 (−0.952 to 0.232)	−0.084	0.23	0.24
Age (years)	2.171 (1.682 to 2.660)	0.612	<0.001	-
Sex (female vs. male)	1.402 (−1.611 to 4.414)	0.063	0.36	-
Model	R^2^ = 0.408; adjusted R^2^ = 0.394; F_(3, 128)_ = 29.4; *p* < 0.001			
Trunk fat mass (kg)				
KIDMED score	−0.196 (−0.524 to 0.131)	−0.078	0.24	0.24
Age (years)	1.384 (1.114 to 1.655)	0.666	<0.001	-
Sex (female vs. male)	0.072 (−1.596 to 1.740)	0.005	0.93	-
Model	R^2^ = 0.472; adjusted R^2^ = 0.459; F_(3, 128)_ = 38.1; *p* < 0.001			
Body fat (%)				
KIDMED score	−0.249 (−0.651 to 0.152)	−0.104	0.22	0.24
Age (years)	0.153 (−0.178 to 0.485)	0.077	0.36	-
Sex (female vs. male)	4.128 (2.084 to 6.171)	0.330	<0.001	-
Model	R^2^ = 0.134; adjusted R^2^ = 0.114; F_(3, 128)_ = 6.6; *p* < 0.001			
Trunk fat (%)				
KIDMED score	−0.261 (−0.692 to 0.169)	−0.100	0.23	0.24
Age (years)	0.732 (0.376 to 1.088)	0.339	<0.001	-
Sex (female vs. male)	1.546 (−0.647 to 3.739)	0.114	0.17	-
Model	R^2^ = 0.154; adjusted R^2^ = 0.134; F_(3, 128)_ = 7.8; *p* < 0.001			

Abbreviations: B—unstandardized regression coefficient; β—standardized regression coefficient; CI—confidence interval; BH—Benjamini–Hochberg; R^2^—coefficient of determination. Sex was coded as 0 = male and 1 = female. Predictors were entered simultaneously using the Enter method. BH correction was applied only to the four KIDMED coefficients, with age and sex included as adjustment variables.

## Data Availability

The data supporting the findings of this study are available from the corresponding author upon reasonable request, subject to applicable ethical and privacy restrictions.

## Supplementary Materials

The following supporting information can be downloaded at https://www.mdpi.com/article/10.3390/nu18213576/s1: Table S1: Anthropometric and body composition parameters according to KIDMED index categories; Table S2: Number (%) of participants who answered “yes” to individual KIDMED questionnaire items according to age group.
