# Supplementary material for: Adherence to the Mediterranean Diet and Body Composition in Children and Adolescents with Obesity: A Cross-Sectional Study
Source: Nutrients. 2026 Oct 29;18(21):3576. doi: 10.3390/nu18213576 (PMC13764788; doi:10.3390/nu18213576)
Supplement: Supplementary file 1 [file nutrients-18-03576-s001.zip › nutrients-4609099-supplementary.pdf]

Supplementary Table S1. Anthropometric and body composition parameters according to KIDMED index categories**.**

	Median (IQR) KIDMED				
	Very low diet quality (≤ 3)	Improvements are needed to adjust intake to Mediterranean patterns (4 - 7)	Optimal diet quality (≥ 8)	*p* value*	*p value (BH)*
	(1)	(2)	(3)		
Age (years)	15 (13 - 17)	13 (11 - 15)	14 (11 - 16)	0.03†	0.69
WHR	0.9 (0.8 - 0.9)	0.9 (0.8 - 0.9)	0.9 (0.8 - 0.9)	0.96	0.96
WHtR	0.57 (0.53 - 0.61)	0.57 (0.53 - 0.62)	0.56 (0.54 - 0.59)	0.70	0.84
Body weight (kg)	87.6 (70.03 - 97.68)	81.7 (67.1 - 95)	78 (58.85 - 99.25)	0.40	0.74
Body height (cm)	166.9 (156.4 - 173.8)	164(149.4 - 172.7)	161.7 (151.9 - 175.2)	0.55	0.74
BMI z-score	2.73 (2.36 - 3.1)	2.87 (2.45 - 3.31)	2.7 (2.26 - 3.28)	0.32	0.74
Body fat (%)	35.45(31.48 - 40.23)	33.9 (31.1 - 38.75)	34.3 (29.1 - 38.15)	0.54	0.74
Fat mass (kg)	29.6 (24.8 - 38.38)	26.3 (20.6 - 36.2)	27.4 (18.9 - 31.85)	0.21	0.74
FFM (kg)	55.1 (44.2 - 65.93)	52.5 (42 - 61.55)	49.8 (42.6 - 66.1)	0.55	0.74
TBW (kg)	38.7 (32.23 - 47.23)	38 (30.55 - 43.8)	36.2 (31.2 - 45.9)	0.53	0.74
Muscle mass (kg)	52.3 (41.93 - 62.7)	49.8 (39.85 - 58.45)	47.3 (40.4 - 62.8)	0.38	0.74
SMI	8.38 (7.47 - 9.15)	8.21 (7.42 - 9.31)	8.27 (7.09 - 9.35)	0.89	0.93
Impedance (ohm)	582 (525 - 621.5)	571 (539 - 622)	579 (538 - 637.5)	0.87	0.93
PhA (°)	5.6 (5.2 - 5.9)	5.6 (5.3 - 6.15)	5.8 (5.25 - 6.1)	0.66	0.84
Trunk fat (%)	32.05 (27.55 - 37.08)	30.2 (25.85 - 34.25)	28.9 (24.6 - 35)	0.32	0.74
Trunk fat mass (kg)	13.95 (10.48 - 19.03)	11.6 (9.05 - 15.8)	12.2 (8.6 - 17.35)	0.15	0.74
Trunk fat-free mass (kg)	30.45 (25.23 - 35.1)	29.2 (22.3 - 33.7)	26.5 (23.55 - 35.85)	0.42	0.74
Trunk muscle mass (kg)	29.05 (24.05 - 33.48)	27.9 (21.05 - 32.1)	25.1 (22.3 - 34.2)	0.40	0.74

Abbreviations: *Kruskal–Wallis test; Conover post hoc test. †Groups (1) and (2) differed significantly before correction for multiple testing (nominal $p < 0.05$). No overall Kruskal–Wallis comparisons remained statistically significant after Benjamini–Hochberg correction. Abbreviations: BH—Benjamini–Hochberg; WHR—waist-to-hip ratio; WHtR—waist-to-height ratio; BMI—body mass index; FFM — Fat-free mass, TBW—total body water; SMI—skeletal muscle index, PhA —phase angle.

Supplementary Table S2. Number (%) of participants who answered "yes" to individual KIDMED questionnaire items according to age group.

KIDMED dietary recommendation	Number (%) of participants			*p*value*
	5 - 9.9 years (*n* = 20)	10 - 19 years (*n* = 112)	Total (*n* = 132)	
Consumes fruit or fruit juice every day	15 (75)	79 (71)	94 (71)	0.69
Consumes a second serving (or more) of fruit every day	11 (55)	45 (40)	56 (42)	0.22
Consumes vegetables (cooked or raw) once a day	16 (80)	80 (71)	96 (73)	0.43
Consumes vegetables (cooked or raw) more than once a day	9 (45)	23 (21)	32 (24)	0.02
Consumes fish at least 2–3 times a week	3 (15)	30 (27)	33 (25)	0.26
Consumes fast food (e.g., hamburgers) more than once a week	2 (10)	34 (30)	36 (27)	0.06
Consumes legumes more than once a week	13 (65)	48 (43)	61 (46)	0.07
Consumes rice or pasta almost daily (at least five times a week)	4 (20)	36 (32)	40 (30)	0.28
Consumes cereals or cereal products (e.g., bread) for breakfast	19 (95)	89 (79)	108 (82)	0.12
Consumes nuts at least 2–3 times a week	7 (35)	44 (39)	51 (39)	0.72
Olive oil is used in the household to prepare the child's meals	17 (85)	100 (89)	117 (89)	0.70
Skips breakfast	8 (40)	47 (42)	55 (42)	0.87
Consumes dairy products (e.g., yogurt or milk) for breakfast	19 (95)	81 (72)	100 (76)	0.04
Consumes pastries or other baked goods for breakfast	8 (40)	55 (49)	63 (48)	0.45
Consumes at least two yogurts and/or cheese (40 g) daily	11 (55)	41 (37)	52 (39)	0.12
Consumes sweets or candy several times a day	8 (40)	47 (42)	55 (42)	0.87

Abbreviations: * Chi-square test. Data are presented as *n* (%), representing participants adhering to each dietary recommendation. Statistical significance was set at $p < 0.05$.
